# Supplementary material for: Superiority of Mesoporous Silica-Based Amorphous Formulations over Spray-Dried Solid Dispersions
Source: Pharmaceutics. 2022 Feb 16;14(2):428. doi: 10.3390/pharmaceutics14020428 (PMC8878785; doi:10.3390/pharmaceutics14020428)
Supplement: Supplementary file 1 [file pharmaceutics-14-00428-s001.zip › pharmaceutics-1601470-supplementary.pdf]

pharmaceutics

Supplementary Materials: Superiority of Mesoporous Silica-Based Amorphous Formulations over Spray-Dried Solid Dispersions

Hongwei Zhang, Minglu Li, Jianmin Li, Anjali Agrawal, Ho-Wah Hui and Demin Liu *

Table S1. Mean assay and standard deviation of the Feno-loaded MS or SDD samples from top, mid-dle and bottom location at initial and 3M accelerated open-dish stage of 40 °C/75% RH.

Sample ID	Fenofibrate Drug Load (%)									
	Initial					3 months at 40 °C/75% RH, open dish				
	Top	Middle	Bottom	Mean	SD	Top	Middle	Bottom	Mean	SD
10%-Feno-MS	10.9	9.4	9.5	9.9	0.8	10.9	10.1	8.5	9.8	1.2
20%-Feno-MS	18.8	22.8	21.0	20.9	2.0	21.5	21.1	20.8	21.1	0.4
30%-Feno-MS	32.6	31.4	33.7	32.6	1.2	32.3	33.8	33.2	33.1	0.8
30%-Feno-SDD-PVP	29.4	25.5	33.2	29.4	3.9	N/A	N/A	N/A	N/A	N/A

SD: Standard deviation.

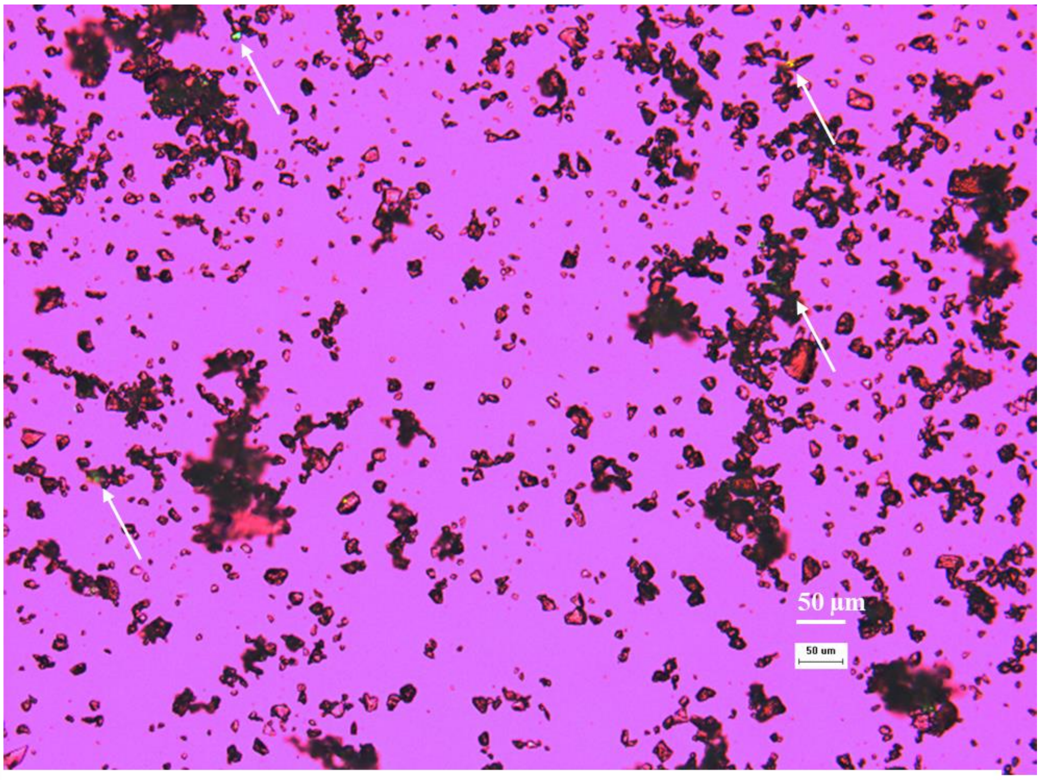

Trial 1: 30% drug load, 50 rpm of paddle stirring speed and 2 mL/min of loading speed for 50 mg/mL of fenofibrate in acetone

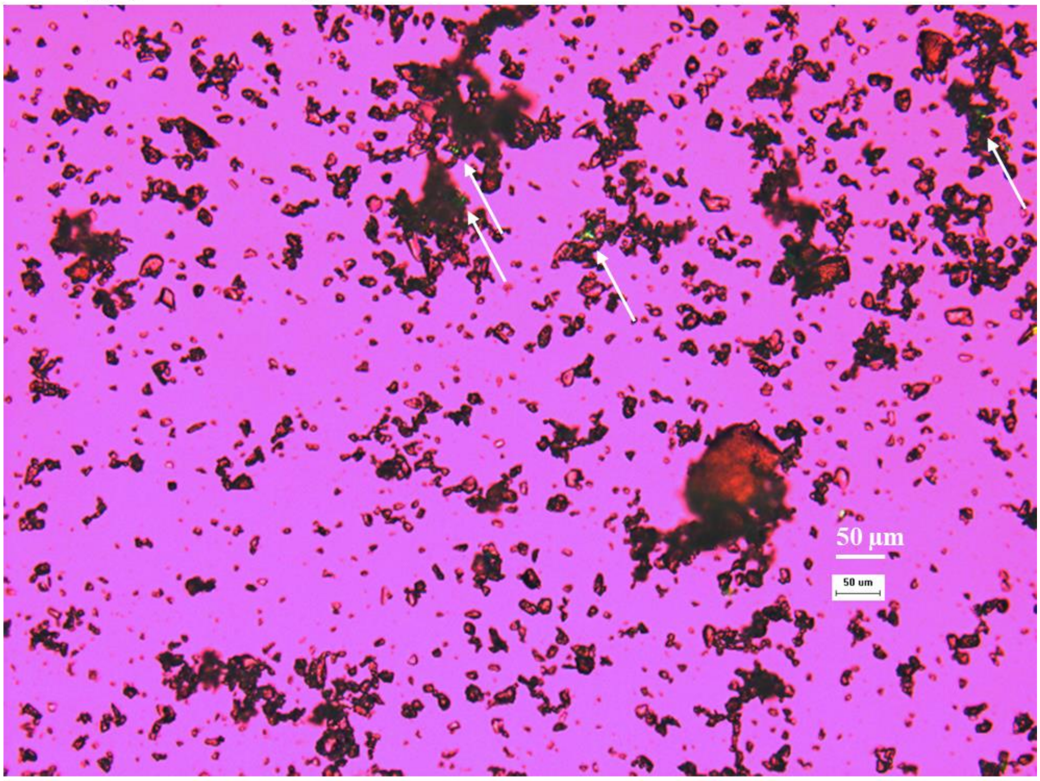

Trial 2: 30% drug load, 100 rpm of paddle stirring speed and 4 mL/min of loading speed for 50 mg/mL of fenofibrate in acetone

Figure S1. PLM images of Feno-MS samples (30% drug load) from preliminary trial experiments. Trial 1: 50 rpm of paddle stirring speed and 2 mL/min of loading speed for 50 mg/mL of fenofibrate in acetone. Trial 2: 100 rpm of paddle stirring speed and 4 mL/min of loading speed for 50 mg/mL of fenofibrate in acetone. Magnification × 100.

Result and Discussion: Multiple recrystallized particles were found by PLM for both trial batches. Agglomeration of wet cake was observed under low paddle stirring speed of 50 rpm or at fast solution loading speed of 4 mL/min, which would increase drug content in local domain and accelerate the recrystallization of fenofibrate outside the silica pores.

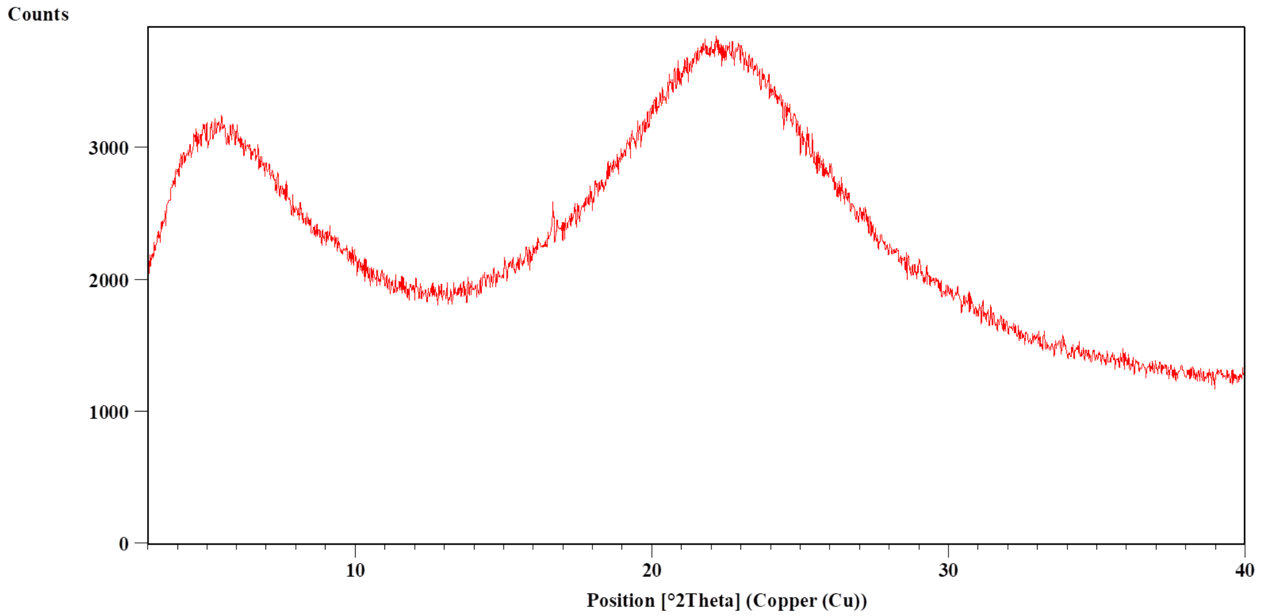

Figure S2. PXRD pattern of Feno-MS sample at 40% drug load.

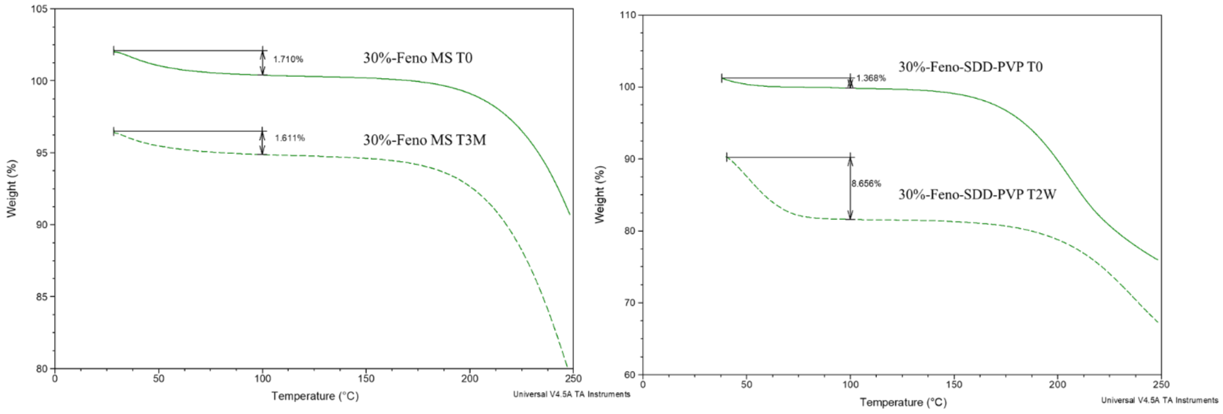

Figure S3. TGA of 30%-Feno MS sample at T0 and T3M after open dish stage of 40 °C/75% RH (left); TGA of 30%-Feno-SDD-PVP sample at T0 and T2W after open dish stage of 40 °C/75% RH (right).
